# Case report: Fully endoscopic microvascular decompression for glossopharyngeal neuralgia

**DOI:** 10.3389/fsurg.2022.1089632

**Published:** 2023-01-06

**Authors:** Haotian Jiang, Dewei Zhou, Pan Wang, Longwei Zeng, Jie Liu, Chao Tang, Gang Zhang, Xiaorong Tan, Nan Wu

**Affiliations:** Department of Neurosurgery, Chongqing General Hospital, Chongqing, China

**Keywords:** endoscopy, microvascular decompression, glossopharyngeal neuralgia, suboccipital retrosigmoid approach, functional neurosurgery

## Abstract

With the advances in endoscopic technology, endoscopy is widely used in many neurosurgical procedures, such as microvascular decompression, which is an effective method to treat glossopharyngeal neuralgia, trigeminal neuralgia, and facial spasm. The purpose of this study was to determine the efficacy of fully endoscopic microvascular decompression in the treatment of glossopharyngeal neuralgia. We managed a patient with glossopharyngeal neuralgia in our department, whose main clinical manifestation was recurrent left ear and facial pain for 3 years. The patient underwent a fully endoscopic microvascular decompression. The pain in the left ear and face was significantly relieved postoperatively, and there was no recurrence at the 6-month follow-up evaluation. We describe a case of glossopharyngeal neuralgia that was successfully treated by fully endoscopic microvascular decompression, which showed that endoscopy has advantages in microvascular decompression, and fully endoscopic microvascular decompression is an effective method for glossopharyngeal neuralgia.

## Introduction

Glossopharyngeal neuralgia (GPN) is a rare cranial nerve compression syndrome that may be caused by the contact or conflict of vessels and nerves. The annual incidence of GPN is 0.2–0.8/100,000 per year (1). The main clinical symptoms of GPN include paroxysmal, temporary, and severe tingling in the distribution of the ninth cranial nerve, such as the root of the tongue, soft palate, tonsils, pharyngeal column, posterior pharyngeal wall, and inner ear (2–5). Therefore, swallowing, coughing, talking, chewing, or yawning induce pain, which severely affects the patient's quality of life (6–11). In addition, the glossopharyngeal nerve is close to the vagus nerve, thus some GPN patients have bradycardia, hypotension, fainting, and even cardiac arrest (12, 13). Currently, the first-line treatment for GPN is drug therapy, including carbamazepine, phenytoin, clonazepam, and gabapentin, of which carbamazepine is the first choice (14, 15). Drug therapy, however, is not always effective for all patients. If the patient's symptoms are not relieved after drug treatment or the patient has drug intolerance, surgical treatment can be considered. The surgical methods available to treat GPN include a percutaneous glossopharyngeal nerve block, microvascular decompression (MVD), gamma knife stereotactic radiosurgery, radiofrequency thermocoagulation, and rhizotomy (16–18). MVD is considered the first-line surgical option for the treatment of GPN (19). When compared with a light microscope, the endoscope has better lighting and vision, thus the surgeon can more effectively visualize the nerves and invading vessels, comprehensively assess the degree of decompression, and reduce the degree of surgical trauma and the associated complications (20–22), thus making the endoscope an important auxiliary tool in patients undergoing MVD. In this study we managed a patient with GPN in our department, the main clinical manifestations of which included recurrent left ear and facial pain for 3 years. Preoperative magnetic resonance imaging of the head showed that the left vertebral artery compressed the posterior group of cranial nerves. The patient underwent fully endoscopic MVD. The pain in the left ear and face was significantly relieved postoperatively, and there was no recurrence at the 6-month follow-up evaluation. Thus, endoscopy has advantages in MVD, and fully endoscopic MVD is a relatively safe, feasible, and effective treatment for GPN.

## Case report

### Patient

**Figure 1:**
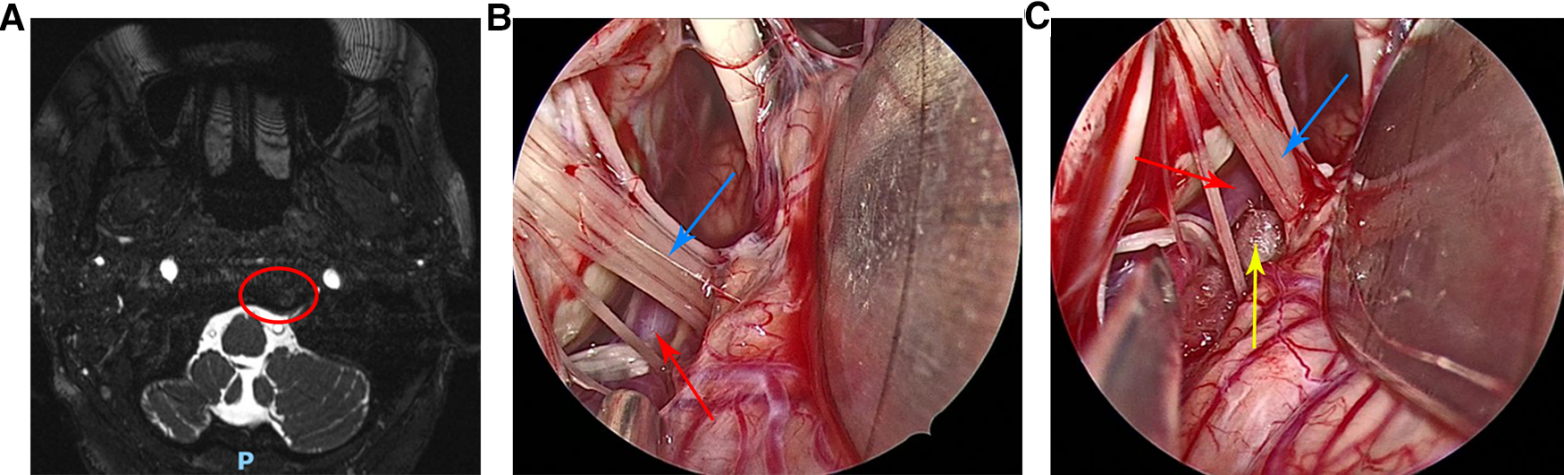
(**A**) Preoperative head magnetic resonance imaging of the patient. (**B**) The left vertebral artery and left glossopharyngeal nerve were clearly visible under the endoscopic view. (**C**) The Teflon pad was placed between the left glossopharyngeal nerve and the left vertebral artery under the endoscopic view. Red circle indicates posterior cranial nerve was compressed by the left vertebral artery, red arrow indicates the left vertebral artery, blue arrow indicates the left glossopharyngeal nerve and the yellow arrow indicates the Teflon pad.

A 57-year-old female was admitted to our hospital in January 2022. The main clinical symptom was paroxysmal, lancinating pain in the left ear and face for approximately 3 years; the pain was induced by cold air. The neurologic examination was normal and the pathologic signs were negative. All laboratory tests, including routine blood, liver and kidney function, and immune and blood coagulation function tests were within normal ranges. The patient had no history of trauma and no family history of genetic diseases. The patient received oral analgesics and acupuncture to the left face, but the effect was not satisfactory. Preoperative magnetic resonance imaging (MRI) of the head showed that the left vertebral artery compressed the posterior group of cranial nerves (Figure 1A). The diagnosis of left GPN was considered based on the patient's clinical manifestations and cranial MRI findings. Due to the increase in severity of pain, intolerance of oral analgesics, and severe impact on the quality of life, the patient underwent fully endoscopic MVD through a suboccipital retrosigmoid approach.

### Surgical procedure

After general anesthesia, the patient was placed in the right lateral decubitus position with the head lowered 15° and rotated 10° to the contralateral side. The neck was slightly forward, the mandible was approximately two transverse fingers away from the sternum, and the mastoid on the operating side was roughly parallel to the operating table in the highest position. First, a vertical incision approximately 5 cm in length was made behind the ear, the skin and muscle were incised, and the base of the mastoid was fully exposed. A bone window approximately 3 cm*3 cm in size was removed by a drill, with the upper edge reaching below the transverse sinus and the lower edge reaching the skull base. Then, the dura mater was exposed and incised, and the arachnoid was incised to slowly release cerebrospinal fluid, so that the cerebellar hemisphere naturally collapsed to create sufficient surgical space. Next, under the endoscope, the arachnoid between the glossopharyngeal nerves was completely separated, and the root of the left glossopharyngeal nerve was fully exposed. Intraoperatively it was seen that the left vertebral artery was in close contact with the glossopharyngeal nerve root (Figure 1B), and the root of the glossopharyngeal nerve was compressed. The offending blood vessels in contact with the glossopharyngeal nerve roots were separated and shifted. A Teflon pad was placed between the offending blood vessel and the glossopharyngeal nerve roots (Figure 1C), and hemostatic gauze covered the surface of the cerebellum. Repeated endoscopy was performed to confirm that no offending vessels were missed, the decompression was adequate, and the position of the Teflon pad was appropriate. Finally, the dura mater was sutured, and the artificial meninges were double-layered to prevent cerebrospinal fluid leakage. The skull defect was repaired with a titanium plate and four screws, and the sutures were closed layer-by-layer.

## Results

After a full endoscopic MVD for GPN, the left ear and facial pain were completely relieved. On the 4th day postoperatively, the patient began coughing repeatedly with sputum production, thus, hypostatic pneumonia was considered. We asked the patient to expectorate more frequently, and treatment with expectorants and antibiotics were initiated. The symptoms improved significantly after treatment. There was no evidence of recurrence at the 6-month follow-up evaluation. Thus, fully endoscopic MVD is an effective method for the treatment of GPN.

## Discussion

GPN is a rare cranial nerve compression syndrome characterized by paroxysmal transient severe pain in the distribution area of the ninth cranial nerve, which severely reduces the quality of life. In 1910, Weisenberg first described a 35-year-old man with severe pain in the glossopharyngeal nerve distribution due to a right cerebellopontine angle tumor compressing the ninth cranial nerve (23). Sicard and Robineau reported three patients with severe pain in the glossopharyngeal nerve distribution for unknown reasons, which was cured by surgical treatment of extracranial nerve avulsion (24). Harris referred to these symptoms as “glossopharyngeal neuralgia” to describe the disorder (25). The first successful intracranial resection for GPN was performed by Dandy in 1927, which was considered the gold standard for the treatment of GPN at the time (9). Until 1977, Laha and Jannetta proposed that MVD could be used to treat GPN and reported three patients with GPN who underwent MVD with good surgical outcomes, concluding that compression of the ninth nerve was the cause of GPN (26). Since that time, surgical methods, such as percutaneous glossopharyngeal nerve block, gamma knife stereotactic radiosurgery, and radiofrequency thermocoagulation, have also been used to treat GPN. Currently, MVD is the most widely used surgical method to relieve blood vessel compression of nerves (15). Patel et al. reviewed >200 GPN patients who underwent MVD, and the overall success rate was >90% (1). Similarly, in a retrospective analysis by Zhao et al. there were 35 patients with GPN who underwent MVD. Thirty-three patients (94.3%) had complete relief of pain immediately after MVD treatment, only 6.6% of the patients relapsed, and none of the patients had long-term surgical complications (13). These results showed that MVD is a safe and effective way to treat GPN. Currently, MVD is generally performed under a microscope; however, the microscope has a direct field of view that makes it difficult for the surgeon to visualize the compressed nerve and the offending blood vessel. Dabey et al. estimated that approximately 23% of dual-offending vessels are lost under a microscope (27). With the rapid advances in endoscopic techniques, the endoscope has been adopted for use in MVD surgery. Compared with a microscope, an endoscope can more accurately detect offending blood vessels. Therefore, according to the type of the optical device, MVD can generally be divided into microscopic MVD, endoscopic-assisted MVD, and fully endoscopic MVD (28–30). Furthermore, depending on whether to use conventional instruments, endoscopic neurosurgery can be divided into pure endoscopic neurosurgery (EN) without conventional instruments, and endoscope-controlled microneurosurgery (ECM) with conventional instruments (31). It is worth noting that the endoscopic MVD in this study has not been further classified and is called “fully endoscopic MVD”. In fact, the “fully endoscopic MVD” mentioned in this study belongs to ECM. Magana and Duntze et al. reported 87 cases of endoscopic-assisted MVD and were of the opinion that an endoscope more accurately identifies the offending vessel (20, 32). At the same time, several studies have reported that an endoscope accurately assesses whether the decompression is sufficient (33, 34). In a large meta-analysis endoscopic MVD was compared with microscopic MVD with respect to the short-term remission, offending blood vessel detection, and long-term remission rates, and concluded that an endoscope is superior to a microscope for MVD (21). In 2002, Jarrahy et al. reported the first GPN patient who was treated by fully endoscopic MVD during which all offending vessels were visualized intraoperatively, and the clinical symptoms were completely relieved after the operation (12). Cureus et al. reported two patients with vagal-glossopharyngeal neuralgia who failed medical therapy and subsequently underwent fully endoscopic MVD (35). The vagal-glossopharyngeal neuralgia symptoms were completely relieved in both patients postoperatively (35). Komatsu et al. also reported how they treated GPN with MVD underwent full endoscopic MVD, and believed that endoscopic MVD is a relevant and minimally invasive method for GPN (36). In the present study we reported a female patient with GPN whose symptoms were refractory to oral analgesics and underwent fully endoscopic MVD. The GPN symptoms were completely relieved and there was no evidence of recurrence at the 6-month follow-up evaluation. These results suggest that fully endoscopic MVD is an effective treatment for GPN. The two key factors for successful MVD surgery are to identify all offending vessels and accurately evaluate decompression. Because the lighting, visual angle, and field of vision provided by an endoscope are superior to a microscope, an endoscope has become an important auxiliary tool in MVD surgery. An endoscope not only reduces the probability of overlooking offending blood vessels, but also determines whether the decompression is sufficient. Moreover, an endoscope also provides surgeons with a more comprehensive view of the relevant anatomic structures, avoids damage to the surrounding brain tissue, blood vessels, and nerves, and reduces the postoperative complications. Therefore, we believe that an endoscope is particularly suitable for MVD. At the same time, we performed a 3*3-cm bone flap intraoperatively. Although a relatively large bone window may increase patient injury, we believed at the time that performing an operation under a relatively large bone window could make the operation more flexible and reduce iatrogenic injuries, and the patient could achieve a better functional outcome. When we perform endoscopic surgery under a small bone window, due to the small space, the instruments used during the surgery cannot reach some angles. The larger bone window increases the flexibility of the operation, protects the brain tissue more effectively, and reduces the risk of damage to surrounding tissues, blood vessels, and nerves. At present, we use a straight skin incision. We suggest that if we use a C-type skin incision and muscle flaps, the integrity of the surrounding soft tissues would be preserved, and the risk of occipital muscle/cutaneous nerve injuries would be decreased, thus obtaining better functional outcomes (37, 38).

## Conclusion

Herein we reported a patient with GPN who was successfully treated using a fully endoscopic MVD, which showed that endoscopy has advantages in performing MVD. Fully endoscopic MVD should be considered safe and effective in the treatment of GPN. We believe that with the development of science and technology and the continuous application and innovation of endoscopy in various fields, endoscopy will play an increasingly important role in neurosurgery in the future.

## Data Availability

The original contributions presented in the study are included in the article/Supplementary Materials, further inquiries can be directed to the corresponding author.
